# Role of Inflammation in the Development of Heart Failure After Myocardial Infarction

**DOI:** 10.31083/RCM49888

**Published:** 2026-09-18

**Authors:** Yachao Li, Mengjie Lei, Zengming Xue

**Affiliations:** ^1^Langfang Core Laboratory of Precision Treatment of Coronary Artery Disease, Langfang People’s Hospital, 065000 Langfang, Hebei, China

**Keywords:** myocardial infarction, heart failure, inflammation

## Abstract

Inflammation plays a key role in the onset and progression of heart failure (HF) after myocardial infarction (MI), which represents one of the leading causes of mortality in patients with cardiovascular disease. This review summarizes the inflammatory mechanisms involved at different stages of HF after MI, including the infiltration and activation of inflammatory cells, the release of inflammatory mediators, and the effects of these mediators on cardiomyocytes, the extracellular matrix, and cardiac function. In addition, this review discusses therapeutic strategies that target inflammatory pathways, providing a theoretical basis for the prevention and treatment of HF after MI.

## 1. Introduction

Myocardial infarction (MI) refers to acute myocardial injury, which is often caused by the severe stenosis or occlusion of the coronary artery and leads to myocardial cell ischemia and hypoxia, thus irreversibly damaging myocardial cells. In the past decade, advances in early reperfusion therapy, especially direct percutaneous coronary intervention (PCI), have been used to save dying myocardial cells and thus greatly improve patient survival rates [1]. MI can trigger an inflammatory response, which leads to the aggregation of immune cells in the infarcted area and the clearance of necrotic cells. Inflammation is followed by ventricular remodeling, a stage that involves cell repair and scar formation. An excessive or sustained inflammatory response can adversely affect heart structure and function, leading to poor ventricular remodeling and promoting the occurrence and development of heart failure (HF). A deep understanding of the role of the inflammatory response in HF after MI is highly important for developing new treatment methods and improving patient prognosis.

## 2. Role of Inflammation in the Acute Phase of MI

### 2.1 Early Infiltration of Inflammatory Cells

Hours after MI, the ischemia and hypoxia of myocardial cells can damage the integrity and barrier function of endothelial cells and thereby enhance vascular permeability and promote leukocyte infiltration. If the ischemic period is long enough, myocardial cells undergo apoptosis and autophagy. Damaged myocardial cells release damage-associated molecular patterns (DAMPs), which activate white blood cells and bind to surviving parenchymal cells and homologous pattern recognition receptors to activate the complement pathways of the innate immune system and hence the inflammatory mediator cascade involving inflammatory cytokines, chemokines, and cell adhesion molecules [2,3]. In addition to being passively released during cell necrosis or matrix damage, DAMPs may also be upregulated and secreted by stressed cardiomyocytes, fibroblasts, and activated white blood cells, synergistically triggering inflammatory responses.

DAMPs initiate inflammation primarily through three core pillars—NF-κB, the NOD-like receptor protein 3 (NLRP3) inflammasome/caspase-1/interleukin-1β (IL-1β) axis, and the Janus kinase/signal transducer and activator of transcription 3 (JAK/STAT3) pathway—which form a signaling cascade network. Nuclear factor kappa B (NF-κB) is one of the most upstream transcription factors that initiate the entire inflammatory cascade, driving the transcription of a large array of proinflammatory genes. These target genes include precursors encoding IL-1β and IL-18 (pro-IL-1β and pro-IL-18, respectively); cytokines such as tumor necrosis factor-α (TNF-α) and interleukin-6 (IL-6); chemokines (e.g., monocyte chemoattractant protein-1 (MCP-1) and IL-8); and adhesion molecules (e.g., intercellular adhesion molecule-1 (ICAM-1) and VCAM-1) that recruit circulating leukocytes. This transcription provides the critical first signal for subsequent inflammasome activation, namely the supply of substrates such as pro-IL-1β. The subsequent assembly of the NLRP3 inflammasome and caspase-1–mediated IL-1β maturation serves as the core engine for pyroptosis and inflammatory amplification. Caspase-1 specifically cleaves pro-IL-1β and pro-IL-18 to produce mature IL-1β and IL-18, both of which exhibit potent proinflammatory activity. IL-1β is a key driver of cardiac rupture, ventricular remodeling, and HF after MI. The JAK/STAT pathway, particularly STAT3 downstream of IL-6 signaling, plays a more complex role after MI, exhibiting both proinflammatory and anti-inflammatory/repair functions. On the one hand, the downstream target genes of STAT3 (such as suppressor of cytokine signaling 3 (SOCS3)) can negatively feedback to inhibit NF-κB and JAK/STAT signaling themselves, thereby limiting inflammatory damage. On the other hand, STAT3 may also synergize with NF-κB under certain circumstances to promote the expression of inflammatory mediators [4].

S100A8/A9, a core member of the DAMP family, belongs to the S100 calcium-binding protein family and is constitutively expressed primarily by myeloid cells (neutrophils and monocytes) [5,6]. Upon inflammatory stimulation, S100A8/A9 is actively secreted extracellularly via a microtubule-dependent mechanism and activates downstream signaling pathways by binding to toll-like receptors and the receptor for advanced glycation end products, thereby amplifying inflammation. Following MI, DAMPs released from necrotic cardiomyocytes stimulate bone marrow myeloid cells, triggering a massive release of S100A8/A9 into the bloodstream. This release drives a large influx of inflammatory cells into cardiac tissue, where they release proteases and reactive oxygen species (ROS), causing secondary damage to the healthy myocardium, increasing the infarct size, and impairing cardiac function. Ma et al. [6] systematically evaluated the value of S100A8/A9 as a predictive biomarker for post-acute MI HF. Vlad et al. [7] demonstrated that short-term blockade limited to the early post-MI period (the first three days) exerts sustained protective effects. The existence of this therapeutic window aligns with the temporal dynamics of the post-MI inflammatory response, where intervention during the inflammation initiation phase is most effective. During the reparative phase of MI (days 3–7), the role of S100A8/A9 is relatively complex. On the one hand, S100A8/A9 promotes the conversion of macrophages toward a reparative phenotype, facilitating necrotic tissue clearance and matrix deposition. On the other hand, the persistent activation of S100A8/A9 may interfere with normal repair processes. Notably, the impact of S100A8/A9 on repair is dose-dependent. Physiological levels of S100A8/A9 are necessary for the macrophage-based clearance of apoptotic cells and fibroblast activation; however, excessively high levels impede repair through the sustained activation of NF-κB and the NLRP3 inflammasome. The core role of S100A9 during the reparative phase lies in regulating the transition of macrophages from a proinflammatory (M1) state to a proreparative (M2) one. In the absence of S100A9, macrophages fail to effectively execute efferocytosis (clearance of extracellular necrotic debris), which leads to the accumulation of necrotic tissue fragments, persistent inflammation, myocardial wall thinning, and structural fragility [5,6,7].

Early revascularization via thrombolysis or direct PCI may also lead to myocardial cell death and myocardial injury during blood flow reperfusion. This phenomenon is known as myocardial reperfusion injury and accounts for 50% of the final MI size [8]. Myocardial tissue damage is further exacerbated because of sudden reoxygenation, ROS production, and complement pathway activation [2,9,10,11,12]. Moreover, the damage attracts more neutrophils to migrate from blood vessels to the infarcted myocardial tissue. After entering the damaged myocardium, the neutrophils play multiple roles, including the phagocytosis of cell debris, extracellular matrix (ECM) degradation by the release of particles containing matrix metalloproteinases, ROS production, and the secretion of factors promoting monocyte chemotaxis. Excessive neutrophil infiltration and/or delayed clearance may exacerbate myocardial injury by prolonging proinflammatory responses [13].

Monocytes are recruited to the infarct area under the action of chemokines and differentiate into macrophages. Early infiltrating macrophages are mainly M1 macrophages, which exhibit high phagocytic and antigen presentation abilities, produce ROS, and secrete large amounts of proinflammatory factors and matrix metalloproteinases, thereby establishing a local proinflammatory microenvironment and amplifying the inflammatory response [14].

### 2.2 Release of Inflammatory Mediators and Their Effects

Cellular transport during inflammation development is controlled by chemical mediators, with aseptic inflammation during acute MI being no exception. The most effective inflammatory mediators are produced from essential polyunsaturated fatty acids. White blood cells recruited to the myocardium are important sources of these mediators, and macrophages are the main subtype of the involved cells [15]. Although the acute phase of inflammation is controlled mainly by prostaglandins and leukotrienes, specialized proresolving mediators (SPMs) are involved in inflammation dissolution [16]. The timely transition of lipid metabolism from leukotriene B4 production to SPM synthesis limits the damage to the myocardium caused by proinflammatory white blood cells and provides effective inflammation resolution. However, the recruited inflammatory cells, such as macrophages and mast cells, release various inflammatory cytokines, including TNF-α, IL-6, histamine, and proteases, which regulate the activity of matrix metalloproteinases and collagen degradation, thus playing a role in the development of myocarditis and fibrosis [17] and leading to myocardial structural instability. In addition, inflammatory mediators can cause endothelial dysfunction and myocardial tissue edema, increase vascular permeability, and affect the normal systolic and diastolic function of the heart.

## 3. Role of Inflammation in the MI Healing Period

In the acute phase of MI, the repair and proliferation phases follow the inflammatory phase, promoting wound healing and appropriate scar formation. The intact myocardium actively participates in late remodeling. Scarring aims to prevent myocardial rupture and partial functional deterioration. This goal is achieved through anti-inflammatory signaling, inflammatory neutrophil consumption, fibroblast proliferation, and granulation tissue deposition [18].

### 3.1 Conversion of Inflammatory Cells

After myocardial healing, a timely shift of the immune system from inflammatory activity to anti-inflammatory activity is required. Neutrophils play a proinflammatory role during the inflammatory phase; however, during the MI healing phase, their clearance from myocardial tissue is considered one of the main signs of inflammation resolution [19]. When matrix metalloproteinases are released to clear necrotic tissue, neutrophils begin to release prolytic lipid mediators to combat inflammatory reactions. Neutrophils can be divided into N1 and N2 subgroups, with CD206-N1 and CD206+N2 neutrophils being inflammatory and anti-inflammatory cells, respectively [20]. In mice, N1 neutrophils dominate in the early stages of MI, whereas N2 neutrophils peak on days 5–7 after MI. In a model of chronic MI, total neutrophil depletion was found to be associated with increased fibrosis and cardiac function deterioration [21]. Similarly, macrophages differentiate into M1 and M2 types under the action of different stimuli. M1 macrophages have an inflammatory phenotype and produce cytokines such as IL-1β, IL-6, and TNF-α, whereas selectively activated M2 macrophages have anti-inflammatory effects and secrete cytokines such as interleukin-10 (IL-10) and transforming growth factor β (TGF-β). IL-10 inhibits the activation of inflammatory cells and the release of inflammatory mediators, reducing the inflammatory response, whereas TGF-β stimulates fibroblast proliferation and collagen synthesis, promotes scar formation in the infarcted area, and contributes to cardiac structural repair and stability. Activated fibroblasts induce cell proliferation, collagen deposition, and angiogenesis [19,22]. However, if the inflammatory response is uncontrolled and M1 macrophages continue to dominate, scarification is hindered, and the risk of heart rupture increases.

### 3.2 Activation of Fibroblasts and ECM Remodeling

The proliferation stage begins after the healing stage, with the loss of proinflammatory signals such as IL-1β and interferon gamma-induced protein 10, allowing cardiac fibroblasts in the border area of MI to differentiate into myofibroblasts [23]. Activated fibroblasts proliferate and synthesize large amounts of ECM components, including collagen I and III. Moderate ECM remodeling is crucial for maintaining the structure and function of the heart; however, excessive ECM deposition can lead to myocardial fibrosis, increasing myocardial stiffness, reducing compliance, and affecting the diastolic function of the heart. In noninfarcted distal myocardium, myocardial fibroblasts may continue to be activated under pressure overload, inducing an early activation of the matrix synthesis pathway, which is associated with cardiac fibrosis and diastolic dysfunction. This process is followed by the activation of matrix degradation signals, chamber dilation, and systolic dysfunction. Moreover, myocardial fibrosis can interfere with electrical signal transmission between myocardial cells, increasing the risk of arrhythmia.

## 4. Chronic Inflammation and Development of HF After MI

### 4.1 Continuous Inflammatory-State Damage to the Myocardium

In the chronic stage of HF after MI, the inflammatory response persists. Preliminary evidence suggests that excessive, sustained, and amplified proinflammatory responses after acute MI may exacerbate left ventricular remodeling through (i) the activation of proteases [24]; (ii) increased cytokine expression, which can induce myocardial apoptosis and inhibit contraction; (iii) increased matrix deposition, which may lead to ventricular stiffening and diastolic dysfunction [25]; and (iv) the activation of myocardial fibroblasts in the infarcted border area, which may cause fibrosis to expand into normal tissue. In a rat model, the expression levels of proinflammatory cytokines (IL-6, TNF-α, and IL-1β) in the myocardium at 8 and 20 weeks after acute myocardial infarction (AMI) were found to be substantially correlated with left ventricular end-diastolic volume. The levels of inflammatory cytokines in the circulation, such as TNF-α and IL-6, continue to increase. These cytokines damage the myocardium through various pathways and can induce myocardial cell hypertrophy, apoptosis, and autophagy abnormalities, leading to a decrease in the number of myocardial cells and myocardial dysfunction. In addition, chronic inflammation can promote the progression of myocardial interstitial fibrosis, further disrupting the normal structure and function of the heart.

In addition to classical apoptotic and autophagic cell damage, pyroptosis and ferroptosis play key roles in myocardial cell necrosis. Pyroptosis is primarily mediated by the inflammasome-caspase-1-GSDMD axis, while ferroptosis is characterized by iron-dependent lipid peroxidation. Both are closely related to myocardial ischemia–reperfusion injury and adverse remodeling after MI [26]. Pyroptosis is a proinflammatory form of programmed cell death. Unlike apoptosis, which involves an intact cell membrane and the orderly packaging of intracellular contents, pyroptosis is characterized by the formation of numerous pores in the cell membrane, which leads to the loss of osmotic pressure regulation, rapid cell swelling and rupture, and the complete release of intracellular inflammatory contents (such as IL-1β, IL-18, and high mobility group box-1) into the extracellular space. It is also a critical link connecting DAMP release and the inflammatory storm. Ferroptosis refers to iron-dependent programmed cell death that is characterized by the accumulation of lipid peroxidation and has clear pathological significance in ischemia–reperfusion injury and post-HF remodeling. During the ischemic phase of AMI, a transient depletion of labile iron pools and compensatory iron accumulation are observed, followed by iron overload during reperfusion. This sequence generates large amounts of ROS via the Fenton reaction, driving ferroptosis [27].

### 4.2 Inflammation-Mediated Myocardial Remodeling and Cardiac Function Deterioration

Large infarctions [28] and substantial initial inflammation activation [29] are strong predictors of late cardiac remodeling and HF in humans. Histopathological studies on HF in patients with chronic ischemic cardiomyopathy revealed an increased abundance of tissue macrophages and T lymphocytes and an enhanced expression of adhesion molecules in endothelial cells. These findings suggest that late cardiac remodeling may be partially due to incomplete myocardial injury, with greater damage after MI and an amplified inflammatory response over time, ultimately leading to the occurrence and development of HF. Fig. 1 describes the roles of different inflammatory factors in the development of HF after MI.

**Fig. 1.**
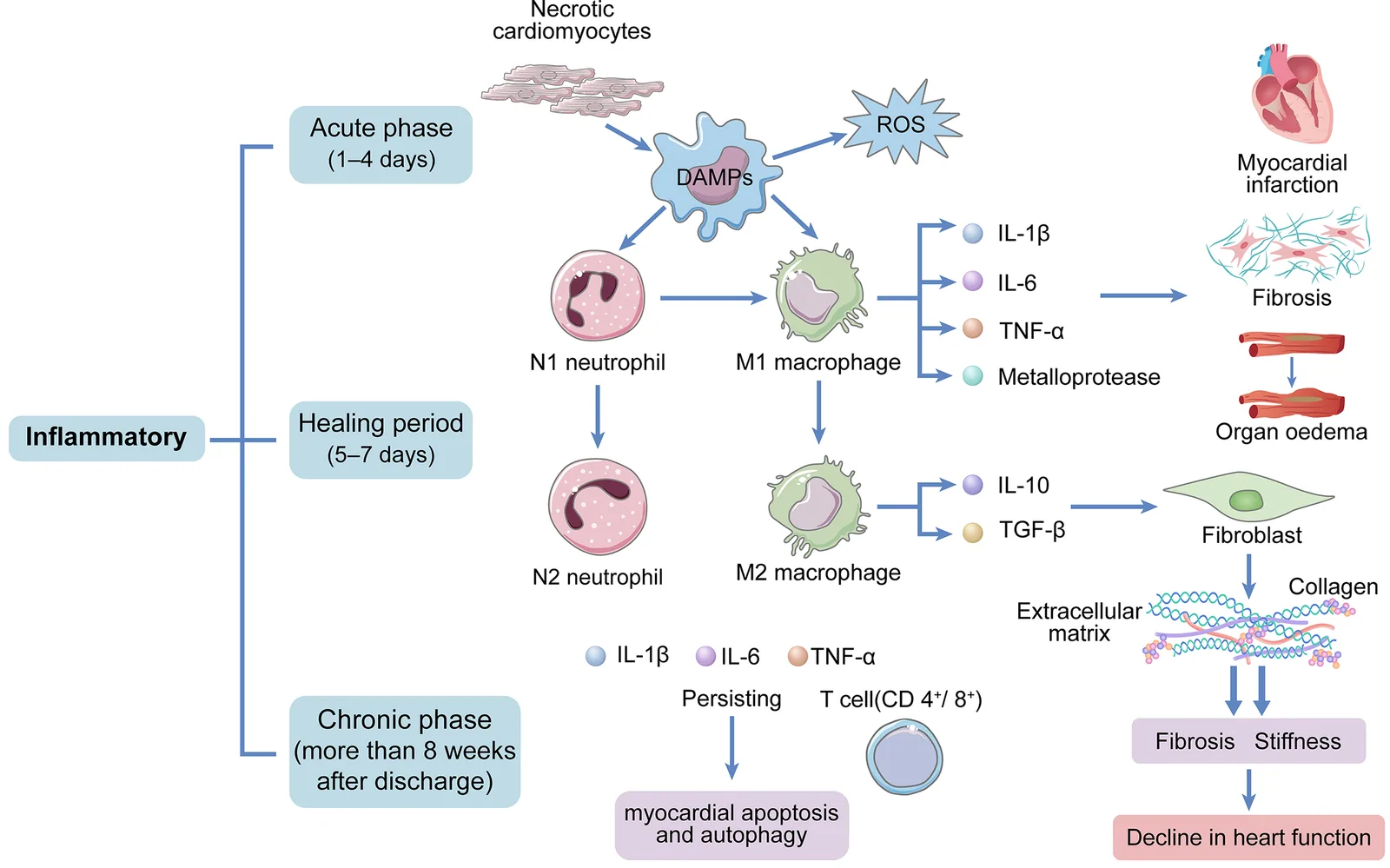
**Roles of different inflammatory factors in the development of heart failure after myocardial infarction**. IL-1β, interleukin-1β; IL-6, interleukin-6; TNF-α, tumor necrosis factor-α; IL-10, interleukin-10; TGF-β, transforming growth factor β.

## 5. Role of Inflammation in Different Types of HF

### 5.1 HF Types

HF after MI can be categorized based on the type of AMI, which is classified into ST-segment elevation myocardial infarction (STEMI) and non-ST-segment elevation myocardial infarction (NSTEMI). HF may develop after either type of MI during hospitalization or after discharge and is categorized into early-onset HF (occurring on admission or during hospitalization) and late-onset HF (occurring after discharge).

In accordance with the differences in the left ventricular ejection fraction (LVEF) and changes after treatment, HF is classified as heart failure with reduced ejection fraction (HFrEF), heart failure with improved ejection fraction (HFimpEF), heart failure with mildly reduced ejection fraction (HFmrEF), and heart failure with preserved ejection fraction (HFpEF) [30,31], with HFpEF having a share of ~50% [32].

### 5.2 Inflammatory Response Characteristics in Patients With Different Types of HF

In patients with HFrEF after MI, the inflammatory response mainly originates from a series of inflammatory cascades caused by the damage, necrosis, or apoptosis of myocardial cells. Collagen replaces dead myocardial cells causing fibrosis, which affects cardiac function. Inflammation in HFpEF patients is mainly caused by a systemic proinflammatory state resulting from comorbidities and affects the myocardial structure and function [33]. Complications include overweight/obesity, diabetes, chronic obstructive pulmonary disease, atrial fibrillation, anemia, and chronic kidney disease. It induces a systemic inflammatory state, leading to coronary microvascular endothelial inflammation and reduced bioavailability of nitric oxide, thus downregulating the activities of cyclic guanosine monophosphate (cGMP) and protein kinase G (PKG).

Inflammation-induced oxidative stress inhibits protein phosphatase 2A or activates the RhoA/Rho kinase pathway via a protein kinase C–dependent mechanism, thereby interfering with PKG activity. The resulting reduction in PKG activity leads to the hypophosphorylation of titin. Hypophosphorylated titin assumes a rigid extended conformation, which increases the passive tension of cardiomyocytes. In patients with HFpEF complicated by conditions such as obesity, chronic inflammation promotes macrophage infiltration into the epicardial adipose tissue, leading to microvascular endothelial inflammation and subsequent coronary microvascular dysfunction. This further reduces myocardial nitric oxide bioavailability and exacerbates abnormalities in energy metabolism, thereby perpetuating the impairment of the cGMP–PKG signaling axis and the adverse mechanical state of titin [34].

Rigid myocardial cells and interstitial fibrosis lead to high diastolic left ventricular stiffness and HF development. The innate immune cells playing a role in HFpEF are mainly neutrophils and macrophages, and the upregulation of endothelial adhesion molecule expression further promotes the infiltration of inflammatory white blood cells into the endothelium, generating endothelial oxidative stress. In summary, in patients with HFpEF, myocardial dysfunction and remodeling are driven by endothelial oxidative stress (Fig. 2), whereas in patients with HFrEF, oxidative stress originates from myocardial cells.

**Fig. 2.**
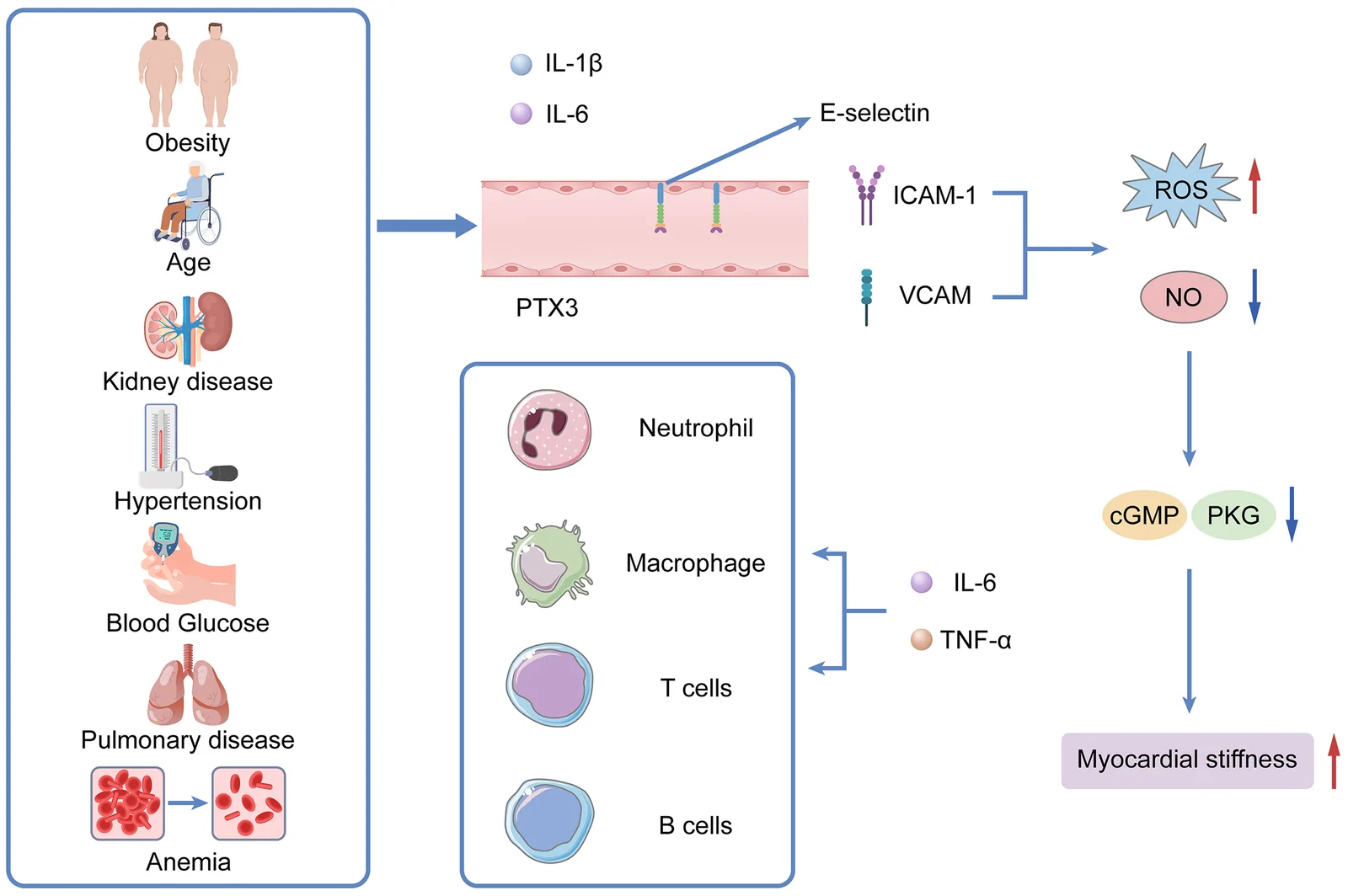
**Roles of inflammatory factors in patients with heart failure with preserved ejection fraction**. ROS, reactive oxygen species; ICAM-1, intercellular adhesion molecule-1; VCAM, vascular cell adhesion molecule; NO, nitric oxide; cGMP, cyclic guanosine monophosphate; PKG, protein kinase G.

## 6. Therapeutic Strategies Based on Inflammatory Targets

### 6.1 Traditional Anti-Inflammatory Drugs

Nonsteroidal anti-inflammatory drugs (NSAIDs), such as aspirin, are widely used in patients after MI. These drugs inhibit the activity of cyclooxygenase (COX), reduce the synthesis of inflammatory mediators such as prostaglandins, alleviate inflammatory reactions [35,36,37], and reduce the incidence of death, stroke, or MI caused by cardiovascular disease while increasing the incidence of adverse reactions such as bleeding [30,38]. However, this anti-inflammatory effect is understood in a broad sense. Aspirin primarily acts by irreversibly acetylating COX-1 in platelets and thereby completely blocks the production of thromboxane A2, a potent vasoconstrictor and platelet aggregation inducer. The purpose of using aspirin is the prevention of secondary thrombus formation at the site of atherosclerotic plaque rupture, which could otherwise lead to MI or ischemic stroke. According to the chronic HF and cardiovascular disease prevention guidelines from the American College of Cardiology/American Heart Association [30] and the European Society of Cardiology [39], nearly all nonaspirin NSAIDs—including selective COX-2 inhibitors and nonselective agents—increase the risk of HF. The underlying mechanisms include sodium and fluid retention (via the inhibition of renal prostaglandin synthesis leading to reduced renal blood flow and the activation of the renin–angiotensin–aldosterone system) and the antagonism of antihypertensive medications, which can raise blood pressure and counteract the therapeutic effects of diuretics, angiotensin-converting enzyme inhibitors, and angiotensin II receptor blockers—standard therapies for HF. Therefore, in clinical guidelines, low-dose aspirin and non-aspirin NSAIDs are treated differently with regard to HF risk.

Statins have long been the drugs of choice for reducing and preventing cardiovascular risk, significantly reducing serum cholesterol levels and the incidence and mortality of cardiovascular diseases [40]. In addition to being used as lipid-lowering agents, statins are used as anti-inflammatory drugs because the mevalonate pathway affects endothelial function, inflammatory response, and coagulation function [41]. Inhibition of proinflammatory cytokine (TNF-α) expression and upregulation of anti-inflammatory cytokine (IL-10) expression during statin therapy have been observed in animal models [42,43]. The JUPITER trial revealed that in the rosuvastatin treatment group but not in the placebo treatment group [44], the incidence of major adverse cardiovascular events associated with reduced levels of high-sensitivity C-reactive protein (hsCRP) significantly decreased, while atherosclerotic plaques were stabilized, and the risk of HF after MI was reduced.

### 6.2 New Anti-Inflammatory Drugs

During the development of arteriosclerosis, the formation of cholesterol crystals activates the NLRP3 inflammasome in inflammatory cells, which produces IL-1β, a key mediator driving plaque progression and instability in the inflammatory cascade [45]. Colchicine is an antagonist of the NLRP3 inflammasome, inhibiting the production of IL-1β. A cohort study [46] including 64 patients with stable coronary heart disease and abnormal hsCRP levels revealed a significant decrease in hsCRP levels in the colchicine treatment group. The results of the Colchicine Cardiovascular Outcomes Trial corroborated the beneficial effects of the early initiation of colchicine after MI, namely the reduced incidence of cardiovascular death, resuscitation from cardiac arrest, MI, stroke, and emergency hospitalization for patients with angina requiring coronary artery reconstruction [47,48].

The DAPA-HF [49] trial marked the beginning of a breakthrough in the use of SGLT2 inhibitors in HF treatment. This randomized, double-blind, placebo-controlled trial enrolled 4744 patients with HFrEF (left ventricular ejection fraction ≤40%). During a median follow-up period of 18.2 months, dapagliflozin reduced the risk of the primary composite endpoint (cardiovascular death or worsening HF) by 26% (HR 0.74, 95% CI 0.65–0.85, *p* < 0.001). This benefit was highly consistent across subgroups with and without diabetes, which strongly indicates that the cardioprotective effects of dapagliflozin are independent of its glucose-lowering action. The nearly simultaneously published results of the empagliflozin outcome trial in patients with chronic heart failure (EMPEROR)-Reduced trial [50] provided cross-validating evidence: Empagliflozin similarly reduced the risk of the primary composite endpoint in patients with HFrEF by 25% (HR 0.75, 95% CI 0.65–0.86, *p* < 0.001). In both trials, the benefit was primarily driven by a significant reduction in HF hospitalization risk, while the reduction in cardiovascular death showed a favorable trend. These data collectively established SGLT2 inhibitors as a standard treatment for HFrEF, positioning them alongside angiotensin receptor–neprilysin inhibitors, beta-blockers, and mineralocorticoid receptor antagonists as one of the new quadruple-cornerstone therapies.

The therapeutic dilemma of HFpEF has long troubled the cardiovascular community. The publication of the results of the EMPEROR-Preserved trial provided positive outcomes. This study enrolled 5988 patients with HF and an ejection fraction of >40% (a considerable proportion had LVEF ≥50%), who were randomly assigned to receive empagliflozin or placebo. Empagliflozin reduced the risk of the primary composite endpoint (cardiovascular death or HF hospitalization) by 21% (HR 0.79, 95% CI 0.69–0.90, *p* < 0.001). This benefit was primarily driven by a reduction in HF hospitalizations and was consistent across different ejection fraction subgroups (>40% to <50% vs. ≥50%) and diabetes status subgroups. The dapagliflozin evaluation to improve the lives of patients with preserved ejection fraction heart failure (DELIVER) trial [51] further supplemented the evidence regarding the role of dapagliflozin in HFpEF treatment. This study enrolled 6263 patients with HF (LVEF > 40%). After a median follow-up period of 2.3 years, dapagliflozin reduced the risk of the primary composite endpoint by 18% (HR 0.82, 95% CI 0.73–0.92, *p* < 0.001). A meta-analysis of EMPEROR-Preserved and DELIVER trials reinforced the robustness of the SGLT2i effect in HFpEF. Currently, SGLT2 inhibitors are the first and only class of HF medications proven effective across the full spectrum of ejection fractions (from ≤40% to ≥50%) [52].

From the perspective of inflammatory mechanisms, elevated levels of inflammatory biomarkers, including C-reactive protein, IL-6, and TNF-α, can be detected in patients with HFrEF or HFpEF [53]. These inflammatory mediators originate not only from the heart itself but also from adipose tissue, skeletal muscle, and circulating immune cells. Inflammation drives the progression of HF through multiple pathways, inducing cardiomyocyte hypertrophy and apoptosis, promoting fibroblast activation and myocardial fibrosis, impairing endothelium-dependent vasodilation, increasing ECM turnover, and ultimately leading to aggravated ventricular remodeling and diastolic dysfunction. Notably, the inflammatory burden in patients with HFpEF generally exceeds that in patients with HFrEF, consistent with the high prevalence of metabolic inflammatory conditions such as obesity, hypertension, and diabetes in the former group. Therefore, targeting inflammatory pathways is considered an important HF treatment strategy, and the anti-inflammatory properties of SGLT2 inhibitors precisely fill this gap.

Current evidence indicates that the anti-inflammatory effects of SGLT2 inhibitors involve multiple interrelated signaling pathways [54]. These agents can inhibit the activation of the NLRP3 inflammasome, which would otherwise trigger the caspase-1-mediated maturation and secretion of IL-1β and interleukin-18 (IL-18) and thereby induce an inflammatory cascade. Various danger signals, such as hyperglycemia, oxidative stress, and cholesterol crystals, can activate the NLRP3 inflammasome. SGLT2 inhibitors inhibit this activation by reducing mitochondrial oxidative stress, regulating potassium efflux, etc., thus curtailing the production of inflammatory mediators at the source. These inhibitors also reduce the generation and release of proinflammatory cytokines. Besides IL-1β and IL-18, classic inflammatory mediators such as TNF-α and IL-6 also show decreasing trends following treatment with SGLT2 inhibitors. These cytokines not only directly impair myocardial contractile function but also induce the expression of adhesion molecules on endothelial cells, promoting transendothelial leukocyte migration and exacerbating local inflammatory responses. Furthermore, SGLT2 inhibitors can modulate the metabolic patterns of immune cells. The switch of macrophages between proinflammatory (M1) and prorepair (M2) phenotypes is highly dependent on metabolic pathway reprogramming. SGLT2 inhibitors promote macrophage polarization towards the M2 phenotype, thereby reducing tissue inflammation and promoting repair [55].

The endothelial protective effects of SGLT2 inhibitors can be translated into improved clinical outcomes in HF through multiple pathways. First, coronary microvascular function improvement increases the myocardial blood flow reserve and alleviates exercise-induced myocardial ischemia and diastolic dysfunction, thereby reducing the risk of HF decompensation. Second, systemic endothelial function protection maintains nitric oxide bioavailability, promotes vasodilation, reduces afterload, improves ventricular–arterial coupling, and enhances cardiac pumping efficiency. Third, the inhibition of the endothelial-to-mesenchymal transition slows the progression of microvascular rarefaction and myocardial fibrosis, which is particularly important for patients with HFpEF, as their pathological hallmarks are increased diastolic stiffness and myocardial fibrosis. Finally, improvement in endothelial function can indirectly reduce cardiomyocyte injury and apoptosis by decreasing the production of oxidative stressors and inflammatory mediators. Thus, through multitarget multilevel vascular protection, SGLT2 inhibitors provide patients with HF with clinical benefits beyond traditional hemodynamic interventions [53].

Inflammation and endothelial dysfunction in HF are not isolated pathological processes but rather mutually reinforcing cause-and-effect vicious cycles. Inflammatory cytokines impair endothelial function by inhibiting nitric oxide synthase and inducing ROS production, while dysfunctional endothelial cells promote the recruitment and activation of inflammatory cells by secreting chemokines and adhesion molecules. The unique value of SGLT2 inhibitors lies in their ability to simultaneously intervene in both key links of this cycle: These agents reduce the inflammatory burden by inhibiting the NLRP3 inflammasome and proinflammatory cytokine production while maintaining vascular integrity by protecting endothelial cell function and inhibiting the endothelial-to-mesenchymal transition [56]. This two-pronged action mode may explain why SGLT2 inhibitors are particularly effective in HFpEF, as the pathological characteristics of the corresponding population are predominantly driven by systemic inflammation and microvascular dysfunction.

Sildenafil, a phosphodiesterase 5 inhibitor (PDE5i), can reduce left ventricular stiffness, improve myocardial diastolic function, enhance rat motility, and increase the VO_2_ peak. These effects are related to the ability of sildenafil to inhibit cGMP degradation and thereby activate PKG. Therefore, the regulatory effect of PDE5is on the cGMP–PKG signaling axis in clinical HFpEF patients can serve as an in-depth research point [57].

Considerable progress has been made in the development of new drugs targeting specific inflammatory targets. For example, clinical trials revealed that IL-1β antagonists (such as canakinumab) exert cardioprotective effects in patients with MI, reducing the levels of inflammatory markers and improving cardiac function [58]. In a randomized trial [59], treatment with another IL-1 inhibitor, anakinra, for three months improved coronary microcirculation by 29% and overall longitudinal strain by 18.7%. The CANTOS trial [58] enrolled patients with a prior history of MI who, despite receiving standard therapy (including statins), had residual inflammatory risk, defined as hsCRP levels of ≥2 mg/L. Patients in the treatment group received canakinumab at doses of 50, 150, or 300 mg administered subcutaneously every three months, with a median follow-up period of 3.7 years. In the group that received the 150 mg dose—the only dose that achieved statistical significance—the risk of the primary endpoint was reduced by 15% (HR 0.85, 95% CI 0.74–0.98, *p* = 0.021). This benefit was primarily driven by a reduction in MI, while no significant differences were observed for stroke or cardiovascular death. The clinical significance of these findings lies in establishing hsCRP as a simple and inexpensive biomarker to identify patients likely to benefit from anti-inflammatory therapy. The CANTOS trial validated the feasibility of anti-inflammatory therapy and revealed the safety trade-offs associated with IL-1β inhibition, namely infectious events. The incidence of fatal infections or sepsis in the canakinumab group was significantly higher than that in the placebo group (0.31 vs. 0.18 per 100 patient-years, *p* = 0.02). Consequently, the CANTOS trial raised a central clinical question of whether an increased risk of infection should be traded for cardiovascular benefit in patients with residual inflammatory risk. This question played a key role in the FDA’s subsequent decision to deny the approval of canakinumab for a cardiovascular indication. Despite this, the CANTOS trial confirmed the feasibility of anti-inflammatory therapy in selected subgroups, and the observation of an hsCRP-dependent response laid an important foundation for the future individualized application of anti-inflammatory therapies (such as colchicine) in cardiovascular diseases.

Tocilizumab competitively inhibits IL-6 receptors and can reduce atherosclerosis and aortic sclerosis and improve endothelial function. The intravenous infusion of tocilizumab reduces perioperative myocardial injury in patients with NSTEMI treated with PCI and decreases the serum levels of high-sensitivity troponin T and C-reactive protein [60].

Another target is TNF-α. TNF-α antagonists etanercept and adalimumab play beneficial roles in atherosclerosis and slow the progression of subclinical diseases by reducing the levels of ICAM-1 and asymmetric dimethylarginine. In addition, the interruption of the TNF-α signaling pathway blocks angiotensin II-induced hypertension [61,62]. However, TNF-α inhibition failed to show efficacy on HF prognosis in the ATTACH and RENAISSANCE trials [63,64] and even exhibited safety signals (increased risk of death and hospitalization) in the high-dose group, which may be attributed to the fact that in the ATTACH trial, the high-dose monoclonal antibody excessively depleted reparative macrophages, leading to a collapse of myocardial repair capacity. Additionally, following TNF-α inhibition, the levels of IL-1β, IL-6, and even TNF-α itself (because of the reduced clearance caused by drug binding) did not consistently decrease in some patients but rather exhibited complex fluctuations. In the RENAISSANCE/RENEWAL trials, although etanercept did not induce cytolysis and had a slightly better safety profile than the monoclonal antibody, it failed to improve clinical endpoints (death or HF hospitalization) and tended to exert neutral or even harmful effects in dose-ranging analyses.

### 6.3 Cell Therapy and Inflammation Regulation

Mesenchymal stem cell (MSC) therapy is a promising approach. The characteristic feature of HF after MI is progressive fibrosis. Fibroblasts and MSCs can differentiate into myofibroblasts that promote fibrosis. MSCs secrete and express platelet-derived growth factor (PDGF) and its receptors. The PDGF signaling pathway in cardiac MSCs (cMSCs) is assumed to promote myofibroblast differentiation and exacerbate left ventricular remodeling and fibrosis after MI. The *in vitro* inhibition of the PDGF signaling pathway can inhibit the differentiation of cMSCs into myofibroblasts, whereas in patients with established ischemic HF, *in vivo* inhibition reduces left ventricular remodeling and dysfunction, myocardial fibrosis, hypertrophy, and inflammation. Therefore, regulating the expression of PDGF receptors in cMSCs may become a novel approach to limiting pathological myocardial fibrosis in patients with HF. Regarding large animal studies, Deshmukh et al. [65] were the first to evaluate the long-term efficacy of PDGF-AB using a porcine model of myocardial ischemia–reperfusion and showed that PDGF-AB achieves sustained cardiac function improvement through vascular protective mechanisms without reducing scar area. On the human research front, the PARIS clinical trial [66], registered in 2025, represents an important step forward in the translation of PDGF signaling research to clinical applications. This study utilized a PET tracer targeting PDGF receptor β to assess its myocardial expression in patients, including those with post-STEMI HF and HFpEF. This technology holds promise for the early identification of patients at high risk of progressing to HF following MI and may serve as a valuable monitoring tool for assessing the efficacy of PDGF/PDGFR-targeted therapies.

### 6.4 Optimal Therapeutic Window for Anti-Inflammatory Therapy

The timing of anti-inflammatory therapy is critical, as inflammation plays a dual role in myocardial injury (such as MI and pressure overload). In the acute phase of MI, inflammation is an indispensable initiating program for tissue repair. The complete suppression of inflammation at this stage (e.g., through the use of high-dose glucocorticoids or broad-spectrum anti-inflammatory drugs) can lead to the delayed clearance of necrotic tissue, insufficient fibroblast activation, and the formation of a weak, thin scar, thereby increasing the risk of cardiac rupture.

However, if the inflammatory response fails to resolve in a timely manner and persists or transitions into low-grade chronic inflammation, a pathological phase ensues. Achieving a dynamic balance from inflammation initiation to resolution is essential. The post-MI inflammatory response is highly time-dependent, and intervention timing determines whether the outcome is protective or harmful. This is where the therapeutic window becomes particularly critical: Intervening at a specific time point can suppress harmful chronic inflammation or excessive fibrosis without interfering with—or even while promoting—the necessary early repair processes. Intervention should be initiated after the third to fifth day post-MI. At this point, after the inflammatory initiation phase is complete, the timely blockade of excessive or persistent inflammatory signals can reduce pathological remodeling without interfering with early repair and can facilitate active inflammation resolution.

However, earlier interventions are also under investigation. For example, S100A9 has been confirmed as a premier biomarker by multiple large-scale clinical cohort studies [67,68,69]. As a therapeutic target, S100A9 is one of the most promising yet most complex candidate molecules in cardiovascular medicine. Blockade therapy targeting S100A9 confers clinical benefits only during the acute inflammatory phase (days 0–3 post-MI). Short-term blockade suppresses the emergency generation of aggressive neutrophils and monocytes from the bone marrow, thereby effectively reducing the inflammatory burden and preventing it from causing permanent structural damage (ventricular wall thinning). Extending the blockade beyond 3–4 days (into the reparative phase) impairs the macrophage-based clearance of necrotic tissue, leading to incomplete healing—a state in which the cardiac scar tissue remains fragile and prone to rupture or dilation. Therefore, the therapeutic window depends on the targeted molecular mechanism. We look forward to the clinical translation of more anti-inflammatory targets with well-defined molecular mechanisms to mitigate the adverse consequences of postinfarction inflammatory responses.

In addition, attention should be paid to the management of chronic inflammation, such as treating comorbidities (e.g., obesity and diabetes) to reduce inflammatory drivers. Achieving this goal reflects the art of balance in immunomodulatory therapy [70] (Fig. 3).

**Fig. 3.**
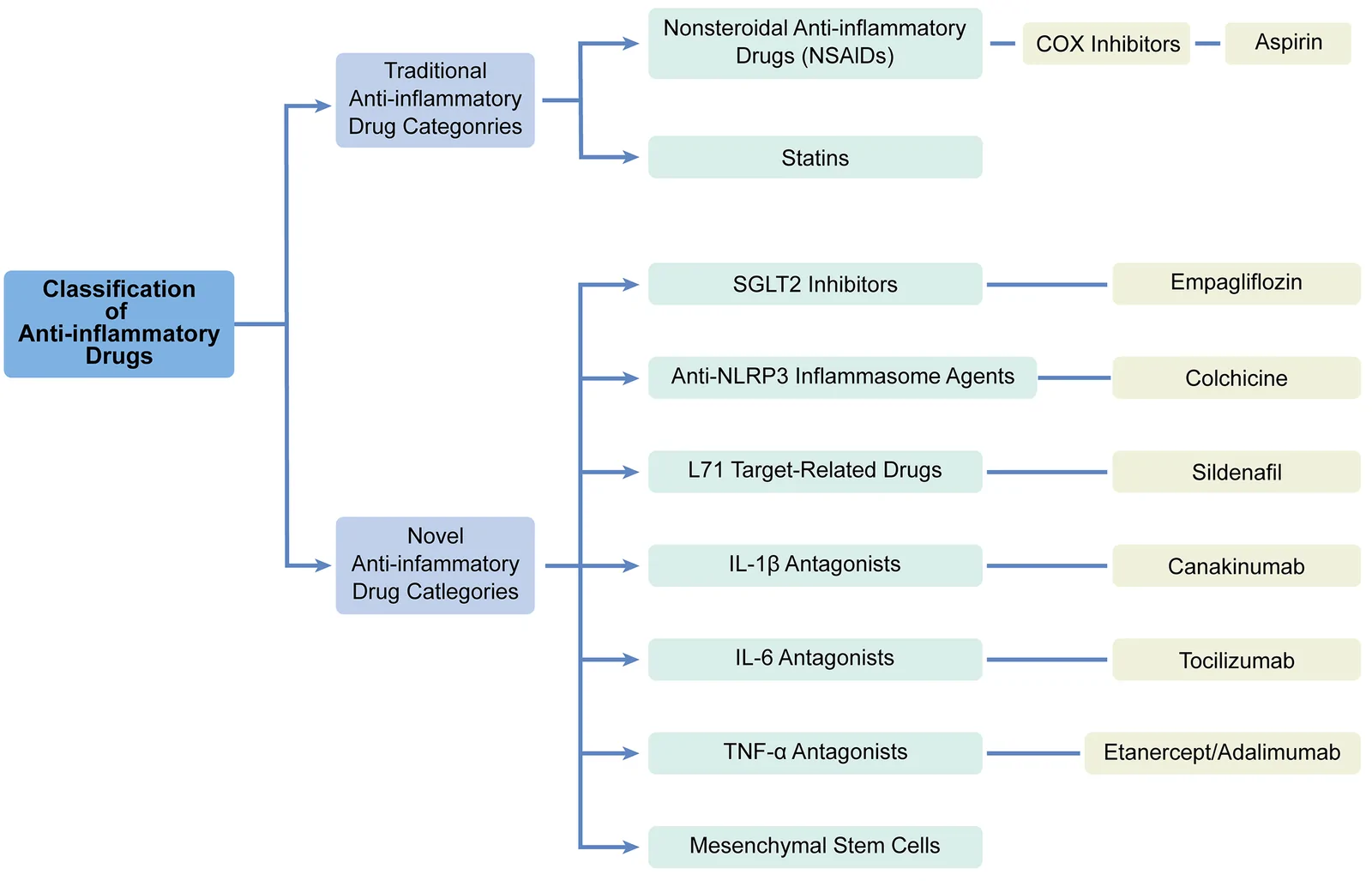
**Classification of anti-inflammatory drugs**.

## 7. Conclusion

An inflammatory response occurs throughout the entire process of HF occurrence and development after MI. In the acute phase of MI, the inflammatory response is initiated to defend the body against myocardial injury, although excessive inflammation can exacerbate this injury. During the healing phase, the inflammatory response is crucial for myocardial repair and scar formation; however, imbalanced inflammation can lead to myocardial fibrosis and structural changes in the heart. In the chronic stage, persistent inflammation is an important driving factor for myocardial remodeling and heart function deterioration. Treatment strategies based on inflammatory targets provide a new direction for the prevention and treatment of HF after MI. However, many problems remain to be solved, such as strategies for accurately regulating the inflammatory response to achieve the best therapeutic effect and the evaluation of the safety and effectiveness of new treatment methods. Future research should explore the molecular mechanisms of the inflammatory response and develop safer and more effective anti-inflammatory treatment methods to improve the prognosis of patients with HF after MI.

## References

[1] Stiermaier T, Eitel I, de Waha S, Pöss J, Fuernau G, Thiele H (2017). Myocardial salvage after primary percutaneous coronary intervention in patients with ST-elevation myocardial infarction presenting early versus late after symptom onset. The International Journal of Cardiovascular Imaging.

[2] Timmers L, Pasterkamp G, de Hoog VC, Arslan F, Appelman Y, de Kleijn DPV (2012). The innate immune response in reperfused myocardium. Cardiovascular Research.

[3] Ghigo A, Franco I, Morello F, Hirsch E (2014). Myocyte signalling in leucocyte recruitment to the heart. Cardiovascular Research.

[4] Zhang Q, Wang L, Wang S, Cheng H, Xu L, Pei G (2022). Signaling pathways and targeted therapy for myocardial infarction. Signal Transduction and Targeted Therapy.

[5] Frangogiannis NG (2019). S100A8/A9 as a therapeutic target in myocardial infarction: cellular mechanisms, molecular interactions, and translational challenges. European Heart Journal.

[6] Ma J, Li Y, Li P, Yang X, Zhu S, Ma K (2024). S100A8/A9 as a prognostic biomarker with causal effects for post-acute myocardial infarction heart failure. Nature Communications.

[7] Vlad ML, Mares RG, Jakobsson G, Manea SA, Lazar AG, Preda MB (2025). Therapeutic S100A8/A9 inhibition reduces NADPH oxidase expression, reactive oxygen species production and NLRP3 inflammasome priming in the ischemic myocardium. European Journal of Pharmacology.

[8] Yellon DM, Hausenloy DJ (2007). Myocardial reperfusion injury. The New England Journal of Medicine.

[9] Frangogiannis NG (2012). Regulation of the inflammatory response in cardiac repair. Circulation Research.

[10] Kain V, Prabhu SD, Halade GV (2014). Inflammation revisited: inflammation versus resolution of inflammation following myocardial infarction. Basic Research in Cardiology.

[11] Eltzschig HK, Eckle T (2011). Ischemia and reperfusion--from mechanism to translation. Nature Medicine.

[12] Vandervelde S, van Amerongen MJ, Tio RA, Petersen AH, van Luyn MJA, Harmsen MC (2006). Increased inflammatory response and neovascularization in reperfused vs. non-reperfused murine myocardial infarction. Cardiovascular Pathology : the Official Journal of the Society for Cardiovascular Pathology.

[13] Prabhu SD, Frangogiannis NG (2016). The Biological Basis for Cardiac Repair After Myocardial Infarction: From Inflammation to Fibrosis. Circulation Research.

[14] Jiao Y, Zhang T, Zhang C, Ji H, Tong X, Xia R (2021). Exosomal miR-30d-5p of neutrophils induces M1 macrophage polarization and primes macrophage pyroptosis in sepsis-related acute lung injury. Critical Care (London, England).

[15] Halade GV, Tourki B (2019). Specialized Pro-resolving Mediators Directs Cardiac Healing and Repair with Activation of Inflammation and Resolution Program in Heart Failure. Advances in Experimental Medicine and Biology.

[16] Serhan CN, Levy BD (2018). Resolvins in inflammation: emergence of the pro-resolving superfamily of mediators. The Journal of Clinical Investigation.

[17] Jaggi AS, Singh M, Sharma A, Singh D, Singh N (2007). Cardioprotective effects of mast cell modulators in ischemia-reperfusion-induced injury in rats. Methods and Findings in Experimental and Clinical Pharmacology.

[18] Peet C, Ivetic A, Bromage DI, Shah AM (2020). Cardiac monocytes and macrophages after myocardial infarction. Cardiovascular Research.

[19] Tourki B, Halade G (2017). Leukocyte diversity in resolving and nonresolving mechanisms of cardiac remodeling. FASEB Journal : Official Publication of the Federation of American Societies for Experimental Biology.

[20] Ma Y, Yabluchanskiy A, Lindsey ML (2013). Neutrophil roles in left ventricular remodeling following myocardial infarction. Fibrogenesis & Tissue Repair.

[21] Horckmans M, Ring L, Duchene J, Santovito D, Schloss MJ, Drechsler M (2017). Neutrophils orchestrate post-myocardial infarction healing by polarizing macrophages towards a reparative phenotype. European Heart Journal.

[22] Beyer M, Mallmann MR, Xue J, Staratschek-Jox A, Vorholt D, Krebs W (2012). High-resolution transcriptome of human macrophages. PloS One.

[23] Shinde AV, Frangogiannis NG (2014). Fibroblasts in myocardial infarction: a role in inflammation and repair. Journal of Molecular and Cellular Cardiology.

[24] Dobaczewski M, Xia Y, Bujak M, Gonzalez-Quesada C, Frangogiannis NG (2010). CCR5 signaling suppresses inflammation and reduces adverse remodeling of the infarcted heart, mediating recruitment of regulatory T cells. The American Journal of Pathology.

[25] Bujak M, Dobaczewski M, Chatila K, Mendoza LH, Li N, Reddy A (2008). Interleukin-1 receptor type I signaling critically regulates infarct healing and cardiac remodeling. The American Journal of Pathology.

[26] Li M, Zhang Y, Hu Y, Qin Y, Zheng Y, Lv S (2026). Mitochondrial homeostasis meets novel programmed cell death: crosstalk mechanisms underlying cardiovascular diseases progression. Cell Communication and Signaling : CCS.

[27] Li T, Wang N, Yi D, Xiao Y, Li X, Shao B (2025). ROS-mediated ferroptosis and pyroptosis in cardiomyocytes: An update. Life Sciences.

[28] Larose E, Rodés-Cabau J, Pibarot P, Rinfret S, Proulx G, Nguyen CM (2010). Predicting late myocardial recovery and outcomes in the early hours of ST-segment elevation myocardial infarction traditional measures compared with microvascular obstruction, salvaged myocardium, and necrosis characteristics by cardiovascular magnetic resonance. Journal of the American College of Cardiology.

[29] van Diepen S, Newby LK, Lopes RD, Stebbins A, Hasselblad V, James S (2013). Prognostic relevance of baseline pro- and anti-inflammatory markers in STEMI: an APEX AMI substudy. International Journal of Cardiology.

[30] Heidenreich PA, Bozkurt B, Aguilar D, Allen LA, Byun JJ, Colvin MM (2022). 2022 AHA/ACC/HFSA Guideline for the management of heart failure: a report of the American College of Cardiology/American Heart Association Joint Committee on Clinical Practice Guidelines. Journal of the American College of Cardiology.

[31] McDonagh TA, Metra M, Adamo M, Gardner RS, Baumbach A, Böhm M (2023). 2023 Focused Update of the 2021 ESC Guidelines for the diagnosis and treatment of acute and chronic heart failure. European Heart Journal.

[32] Mishra S, Kass DA (2021). Cellular and molecular pathobiology of heart failure with preserved ejection fraction. Nature Reviews. Cardiology.

[33] Paulus WJ, Tschöpe C (2013). A novel paradigm for heart failure with preserved ejection fraction: comorbidities drive myocardial dysfunction and remodeling through coronary microvascular endothelial inflammation. Journal of the American College of Cardiology.

[34] Fayyaz AU, Eltony M, Prokop LJ, Koepp KE, Borlaug BA, Dasari S (2025). Pathophysiological insights into HFpEF from studies of human cardiac tissue. Nature Reviews. Cardiology.

[35] Hybiak J, Broniarek I, Kiryczyński G, Los LD, Rosik J, Machaj F (2020). Aspirin and its pleiotropic application. European Journal of Pharmacology.

[36] Baigent C, Blackwell L, Collins R, Emberson J, Godwin J, Antithrombotic Trialists' (ATT) Collaboration (2009). Aspirin in the primary and secondary prevention of vascular disease: collaborative meta-analysis of individual participant data from randomised trials. Lancet (London, England).

[37] Santilli F, Rocca B, De Cristofaro R, Lattanzio S, Pietrangelo L, Habib A (2009). Platelet cyclooxygenase inhibition by low-dose aspirin is not reflected consistently by platelet function assays: implications for aspirin "resistance". Journal of the American College of Cardiology.

[38] Connolly SJ, Eikelboom JW, Bosch J, Dagenais G, Dyal L, Lanas F (2018). Rivaroxaban with or without aspirin in patients with stable coronary artery disease: an international, randomised, double-blind, placebo-controlled trial. The Lancet.

[39] McDonagh TA, Metra M, Adamo M, Gardner RS, Baumbach A, Böhm M (2021). 2021 ESC Guidelines for the diagnosis and treatment of acute and chronic heart failure. European Heart Journal.

[40] Almeida SO, Budoff M (2019). Effect of statins on atherosclerotic plaque. Trends in Cardiovascular Medicine.

[41] Sakellarios AI, Fotiadis DI (2019). Editorial commentary: The pleiotropic effect of statins on the atherosclerotic plaque and coronary heart disease. Trends in Cardiovascular Medicine.

[42] Stumpf C, Petzi S, Seybold K, Wasmeier G, Arnold M, Raaz D (2009). Atorvastatin enhances interleukin-10 levels and improves cardiac function in rats after acute myocardial infarction. Clinical Science (London, England : 1979).

[43] Shen Y, Wu H, Wang C, Shao H, Huang H, Jing H (2010). Simvastatin attenuates cardiopulmonary bypass-induced myocardial inflammatory injury in rats by activating peroxisome proliferator-activated receptor γ. European Journal of Pharmacology.

[44] Ridker PM, Danielson E, Fonseca FAH, Genest J, Gotto AM, Kastelein JJP (2008). Rosuvastatin to prevent vascular events in men and women with elevated C-reactive protein. The New England Journal of Medicine.

[45] Tardif JC, Kouz S, Waters DD, Bertrand OF, Diaz R, Maggioni AP (2019). Efficacy and Safety of Low-Dose Colchicine after Myocardial Infarction. The New England Journal of Medicine.

[46] Nidorf M, Thompson PL (2007). Effect of colchicine (0.5 mg twice daily) on high-sensitivity C-reactive protein independent of aspirin and atorvastatin in patients with stable coronary artery disease. The American Journal of Cardiology.

[47] Bouabdallaoui N, Tardif JC, Waters DD, Pinto FJ, Maggioni AP, Diaz R (2020). Time-to-treatment initiation of colchicine and cardiovascular outcomes after myocardial infarction in the Colchicine Cardiovascular Outcomes Trial (COLCOT). European Heart Journal.

[48] Nidorf SM, Thompson PL (2019). Why Colchicine Should Be Considered for Secondary Prevention of Atherosclerosis: An Overview. Clinical Therapeutics.

[49] Kaplinsky E (2020). DAPA-HF trial: dapagliflozin evolves from a glucose-lowering agent to a therapy for heart failure. Drugs in Context.

[50] Böhm M, Butler J, Coats A, Lauder L, Mahfoud F, Filippatos G (2025). Empagliflozin in resistant hypertension and heart failure with preserved ejection fraction: the EMPEROR-Preserved trial. European Heart Journal.

[51] Solomon SD, McMurray JJ, Claggett B, de Boer RA, DeMets D, Hernandez AF (2022). Dapagliflozin in Heart Failure with Mildly Reduced or Preserved Ejection Fraction. New England Journal of Medicine.

[52] Robert A, Demetrio Sharp D, Jennifer L C, Mahyar P (2023). SGLT-2 INHIBITORS ARE EFFICACIOUS IN HIGH-RISK SUBGROUPS OF PATIENTS WITH HEART FAILURE WITH MILDLY REDUCED OR PRESERVED EJECTION FRACTION. Journal of the American College of Cardiology.

[53] Anker SD, Butler J, Filippatos GS, Jamal W, Salsali A, Schnee J (2019). Evaluation of the effects of sodium-glucose co-transporter 2 inhibition with empagliflozin on morbidity and mortality in patients with chronic heart failure and a preserved ejection fraction: rationale for and design of the EMPEROR-Preserved Trial. European Journal of Heart Failure.

[54] Yoo TT, Baek IH, Stoletniy L, Hilliard A, Sakr A, Doycheva D (2025). Impact of sodium-glucose transport protein-2 (SGLT2) inhibitors on the inflammasome pathway in acute myocardial infarction in type 2 diabetes mellitus: a comprehensive review. Cardiovascular Diabetology.

[55] Checa-Ros A, Okojie OJ, D'Marco L (2025). SGLT2 Inhibitors: Multifaceted Therapeutic Agents in Cardiometabolic and Renal Diseases. Metabolites.

[56] De Lorenzi AB, Kaplinsky E, Zambrano MR, Chaume LT, Rosas JM (2023). Emerging concepts in heart failure management and treatment: focus on SGLT2 inhibitors in heart failure with preserved ejection fraction. Drugs in Context.

[57] Leite S, Moreira-Costa L, Cerqueira R, Sousa-Mendes C, Angélico-Gonçalves A, Fontoura D (2021). Chronic Sildenafil Therapy in the ZSF1 Obese Rat Model of Metabolic Syndrome and Heart Failure With Preserved Ejection Fraction. Journal of Cardiovascular Pharmacology and Therapeutics.

[58] Ridker PM, Everett BM, Thuren T, MacFadyen JG, Chang WH, Ballantyne C (2017). Antiinflammatory Therapy with Canakinumab for Atherosclerotic Disease. The New England Journal of Medicine.

[59] Ikonomidis I, Pavlidis G, Katsimbri P, Andreadou I, Triantafyllidi H, Tsoumani M (2019). Differential effects of inhibition of interleukin 1 and 6 on myocardial, coronary and vascular function. Clinical Research in Cardiology : Official Journal of the German Cardiac Society.

[60] Kleveland O, Kunszt G, Bratlie M, Ueland T, Broch K, Holte E (2016). Effect of a single dose of the interleukin-6 receptor antagonist tocilizumab on inflammation and troponin T release in patients with non-ST-elevation myocardial infarction: a double-blind, randomized, placebo-controlled phase 2 trial. European Heart Journal.

[61] Bergström U, Jovinge S, Persson J, Jacobsson LTH, Turesson C (2018). Effects of Treatment with Adalimumab on Blood Lipid Levels and Atherosclerosis in Patients with Rheumatoid Arthritis. Current Therapeutic Research, Clinical and Experimental.

[62] Chen XY, Yan BX, Man XY (2020). TNFα inhibitor may be effective for severe COVID-19: learning from toxic epidermal necrolysis. Therapeutic Advances in Respiratory Disease.

[63] Chung ES, Packer M, Lo KH, Fasanmade AA, Willerson JT, Anti-TNF Therapy Against Congestive Heart Failure Investigators (2003). Randomized, double-blind, placebo-controlled, pilot trial of infliximab, a chimeric monoclonal antibody to tumor necrosis factor-alpha, in patients with moderate-to-severe heart failure: results of the anti-TNF Therapy Against Congestive Heart Failure (ATTACH) trial. Circulation.

[64] Mann DL, McMurray JJV, Packer M, Swedberg K, Borer JS, Colucci WS (2004). Targeted anticytokine therapy in patients with chronic heart failure: results of the Randomized Etanercept Worldwide Evaluation (RENEWAL). Circulation.

[65] Deshmukh T, Hume RD, Chen S, Igoor S, Foster SL, Barry T (2025). Platelet derived growth factor-AB modulates post-infarct myocardium leading to extended improvement in cardiac function. NPJ Regenerative Medicine.

[66] ClinicalTrials.gov (2025). Platelet Derived Growth Factor Receptor ß (PDGFRß) Imaging in Cardiac Fibrosis (PARIS). https://clinicaltrials.gov/study/NCT06956560?cond=NCT06956560&viewType=Card&rank=1.

[67] Marinković G, Grauen Larsen H, Yndigegn T, Szabo IA, Mares RG, de Camp L (2019). Inhibition of pro-inflammatory myeloid cell responses by short-term S100A9 blockade improves cardiac function after myocardial infarction. European Heart Journal.

[68] Yu Y, Shi H, Wang Y, Yu Y, Chen R (2024). A pilot study of S100A4, S100A8/A9, and S100A12 in dilated cardiomyopathy: novel biomarkers for diagnosis or prognosis?. ESC Heart Failure.

[69] Volz HC, Laohachewin D, Seidel C, Lasitschka F, Keilbach K, Wienbrandt AR (2012). S100A8/A9 aggravates post-ischemic heart failure through activation of RAGE-dependent NF-κB signaling. Basic Research in Cardiology.

[70] Imbesi A, Greco A, Spagnolo M, Laudani C, Raffo C, Finocchiaro S (2025). Targeting Inflammation After Acute Myocardial Infarction. Journal of the American College of Cardiology.

